# One Plus One Makes Three (for Social Networks)

**DOI:** 10.1371/journal.pone.0034740

**Published:** 2012-04-06

**Authors:** Emöke-Ágnes Horvát, Michael Hanselmann, Fred A. Hamprecht, Katharina A. Zweig

**Affiliations:** 1 Interdisciplinary Center for Scientific Computing (IWR), University of Heidelberg, Heidelberg, Germany; 2 Heidelberg Collaboratory for Image Processing (HCI), University of Heidelberg, Heidelberg, Germany; 3 Marsilius Kolleg, University of Heidelberg, Heidelberg, Germany; Universitat Rovira i Virgili, Spain

## Abstract

Members of social network platforms often choose to reveal private information, and thus sacrifice some of their privacy, in exchange for the manifold opportunities and amenities offered by such platforms. In this article, we show that the seemingly innocuous combination of knowledge of confirmed contacts between members on the one hand and their email contacts to non-members on the other hand provides enough information to deduce a substantial proportion of relationships between *non*-members. Using machine learning we achieve an area under the (receiver operating characteristic) curve (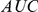) of at least 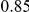 for predicting whether two non-members known by the same member are connected or not, even for conservative estimates of the overall proportion of members, and the proportion of members disclosing their contacts.

## Introduction

Some individuals prefer to keep intimate details such as their political preferences or sexual orientation private. Recent results suggest that such details can nonetheless be inferred with high probability if a sufficient number of confirmed contacts in a social network chooses to reveal *their* details [1]–[4]. As a consequence, some of the more circumspect choose to stay away from social network platforms such as Facebook in the belief that this will help protect their privacy. In this article, we show that such an assumption is no longer valid: with the help of machine learning, social network operators can make predictions regarding the acquaintance or lack thereof between two *non-*members with a high rate of success. To our knowledge these are the first results on the potential of social network platforms to infer relationships between *non*-members.

Inference of undisclosed, unobserved, or future contacts (the “edges”) between people or agents (the “nodes”) is known as the “link prediction” problem [5]–[7]. It is a difficult problem mainly because the imbalance between possible and realized future edges is extremely high in most cases [8], [9]. In contrast, the prediction of some properties of given links, e.g. the sign of the weight on a link [10] is simpler because the problem is typically more balanced. Link prediction was mostly approached with unsupervised [11] and recently also with supervised learning methods [12]–[14]. Inference was done both using solely structural measures based on the network topology [15], [16] but also by additionally taking into account the nodes’ attributes [17]–[19]. The most common setting of the link prediction problem is, given an evolving network at an early stage, to predict newly acquired edges at a later stage. The success of link prediction has usually been estimated by cross-validation within the same network [20], [21]. This typically implies a dependence between training and test data and, hence, an overly optimistic estimate of the accuracy of an algorithm. To our knowledge, we present the first link prediction work where learning and testing are performed on entirely independent networks.

## Methods

### The Problem

**Figure 1:**
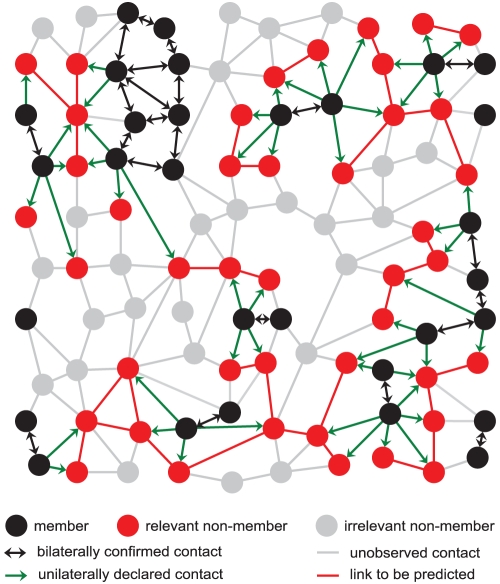
Definitions and examples. Any social network platform divides society into two sets: the set of members 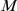 (black nodes) and of non-members 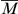. In our toy example 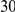 of 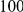 individuals, i.e. a fraction of 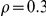, are members. The relevant subset 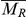 of non-members (red nodes) that are in contact with at least one member is distinguished from other non-members (gray nodes). 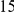 of the 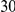 members, i.e., a fraction of 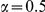, have disclosed their outside social contacts. The knowledge of the set of edges 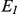 between members (black, bi-directed) and the set of edges 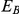 (green) to non-members is enough to infer a substantial fraction of edges between non-members (red edges).

All members of society can be seen as nodes in an unobservable social graph. This latent social graph is dynamic and extremely complex, with edges of widely differing quality (two people may be kindred, or engaged, or work together, they may like or dislike each other, etc.). From the point of view of a social network platform like Facebook, the set of all people can be divided into a fraction 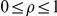 of members and 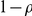 of non-members. The multi-faceted relationships between people are much simplified, in an extreme case into mere binary form: two members may declare a “friendship” which is then represented by an edge in the set 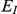. In reality, social networks typically have access to more information that allows to estimate the quality of an edge (its strength, its asymmetry, etc.) especially if they integrate a messaging service. Additionally, a fraction 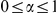 of all platform members may also share their contacts to non-members, e.g., by uploading their email address book (Figure 1). Social network platforms then have direct access to two different sets of relationships: on the one hand, the mutually confirmed contacts between platform members (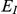); and on the other hand, their members’ unilateral declarations of their acquaintance with non-members (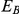). The edges in both 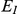 and 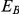 are an abstraction and a subset of the edges in the latent social graph. The central question of this article is to what extent the acquaintance of two people who are *both* non-members can be predicted.

For the very reason that the latent social graph is fundamentally unobservable, both we and a social network operator with similar aims as described here needs to impute the missing information to admit a machine learning procedure. The approach we choose is to use the *observed* part of a social network – say, the Facebook network of all students at a given university – and presume it represents the *complete* (and unobservable) social graph of a hypothetical community. In other words, the edges in this social graph are considered the ground truth. We then proceed to partition this community into a set of members and non-members by a number of member recruitment models outlined below which represent a broad range of potential strategies by which people choose to become members. Finally, we predict the existence or otherwise of an edge between any two non-members and evaluate the accuracy of these predictions with respect to the ground truth.

### Ground Truth Imputation

**Figure 2:**
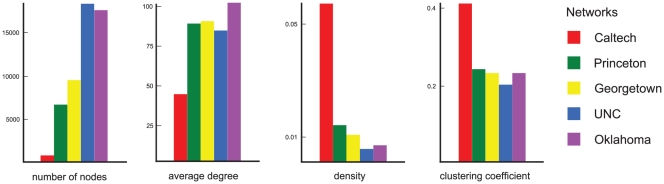
Comparison of basic network analytic statistics of the five data sets obtained from Traud et al. [**22**].

In line with what would be available to a social network operator, we use real social networks, in this case real-world Facebook friendship networks representing the students from five different US universities [22]. Figure 2 shows a comparison of their number 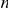 of members, their average degree (the average number of friends a member has), their density 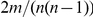 where 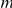 is the number of friends, and their average clustering coefficient (the average probability that two friends of a member are friends themselves [23]).

The ground truth imputation comprises three steps. In the first step the platform penetration percentage 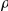 is modeled. The percentage of members in a given population varies strongly with the type of social network platform and the social community of interest. According to Facebook, it had more than 800 million active users in November 2011 [24], while the number of internet users worldwide is estimated at over 2 billion [25]. Thus, roughly 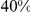 of all internet users are active Facebook members. Around 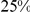 of all Facebook users are US citizens. Assuming that each Facebook account represents one individual, we can estimate that over 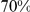 of all North American internet users are members of Facebook. For certain social strata the percentage of Facebook users is known exactly: one study showed that already back in 2005 over 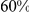 of the undergraduates of the Carnegie Mellon University were members of the platform [26]. Later, in 2009 around 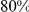 of all interviewed students of the University of Illinois of Chicago [27] and 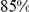 of a polled contemporary Canadian sample [28] were Facebook members. The platform penetration parameter 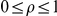 thus reflects different membership densities and allows to model different social network platforms and their acceptance in different communities.

**Figure 3:**
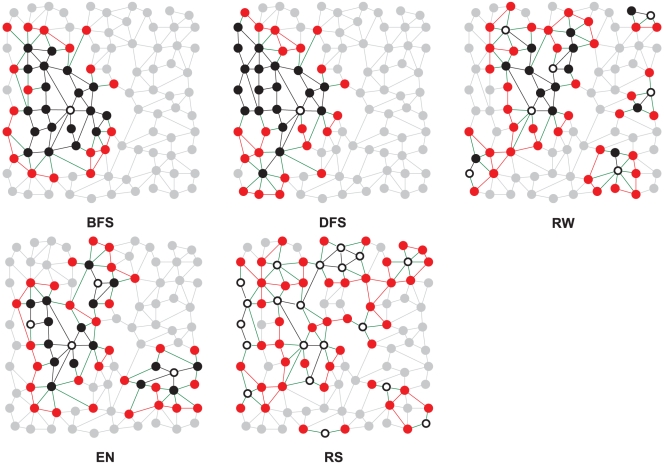
Membership propagation in a toy example according to different propagation models. Note that real social networks exhibit more long-range edges. Examples for the platform penetration value 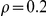 show the nodes from which the propagation started (black nodes with white core). Other members are marked black and relevant non-members red; for ease of reading arrows are not displayed, but black edges are bidirectional while green edges point from black to red nodes. With BFS and DFS the network is explored starting from one node (denoted by a white circle); with RW and EN there are more nodes from which the propagation is launched; and finally, for RS all selected nodes can be seen as starting nodes.

Given a real Facebook friendship network and a choice of 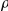, the second step of the ground truth imputation is to partition the nodes of the network into members and non-members. For this we need models for how people choose to become members of a platform which we call *member recruitment models*. An analysis of the evolution of online social networks [29] suggests that a network platform recruits its members through a mixture of online mediated invitations by friends who already are members, and independent decisions by individuals who are not yet friends of a member. Since the actual member recruitment process is unknown and probably also depends on the group of people that is considered (e.g. college students vs. employees), we have emulated the growth of social network platforms using processes ranging from strongly dependent to purely independent decisions. All models start with labeling a node chosen uniformly at random as the first member. Strongly dependent decisions are modeled by processes in which only people who know at least one member will join the network. In a breadth first search (BFS) model all friends of the first member are labeled as members after which all *their* friends are labeled and so on. In a depth first search (DFS) model a randomly chosen friend of the first member joins the platform after which a randomly chosen friend of the new member joins and so on recursively. Less dependent decisions are modeled by a random walk (RW) which is restarted from a new node as soon as a friend of a new member is chosen which is already a member. The ego networks selection (EN) model joins the independent decision of some randomly chosen seed members with the dependent decision of their direct friends. Purely random selection of members (RS) is based entirely on independent decisions modeled by the random selection of a set of members. These member recruitment models are described in more detail in Text S1. For an analysis of the structural properties of the partitions obtained with different member recruitment models see Figures S1, S2, and S3. Figure 3 shows the resulting partitions of a toy graph under all five models. We show below that our main findings are robust with respect to the specific choice of the member recruitment model.

In the third step, a so-called disclosure parameter 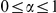 is chosen to model the probability with which a member opens her email address book to the platform, e.g. through revealing her email contacts. We consider that a member who revealed her email contact list shared thereby *all* of her contacts justified by the feature of the platform allowing for easy automatic uploading of the entire email address book. 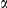 governs the fraction of connections between the member and non-member sets. As such, it is a key ingredient of the ground truth imputation.

Given an underlying graph 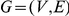 with nodes 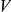 and edges 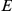, the simulated member recruitment model results in a subset of nodes 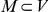 that are considered members, and a set of non-members 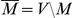. We will only focus on the set 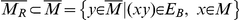 of *relevant* non-members whose email address has been disclosed by at least one member (see Figure 1). These node sets induce the edge sets 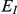 and 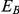 as defined above, and additionally the edges between non-members, which are not directly accessible to the platform, and are at the core of our interest and prediction efforts. Let 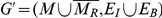 denote the new graph containing all the structural information that is assumed to be known by a given social network platform (black and red nodes, and black and green edges in Figure 1).

The ground truth imputation is thus determined by the choice of the percentage of individuals 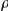 deciding to become members of the social network, the member recruitment model (BFS, DFS, RW, EN, RS), and the choice of 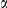, the propensity of members to disclose their contacts with non-members.

### Feature Extraction

**Figure 4:**
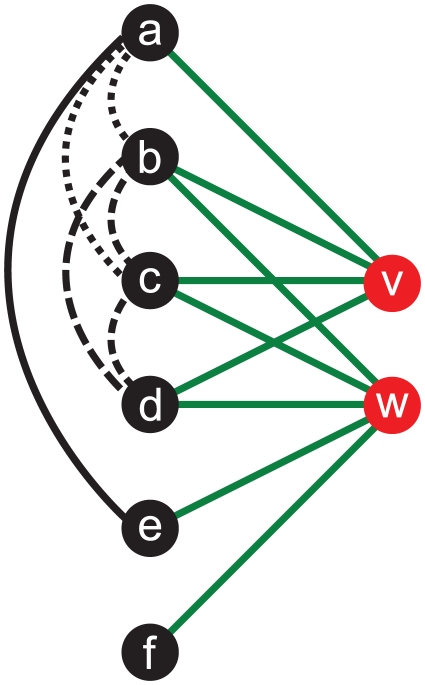
Features based on different edge sets between the exclusive, joint, and common neighborhoods of *v* and *w*. All left-hand nodes belong to the joint neighborhood of 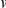 and 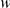. 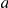 is exclusive to 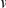, while 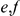 are exclusive to 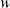, and 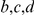 are common neighbors of both. Our features comprise the absolute number of edges between common neighbors (black, dashed edges), exclusive neighbors (black, straight edge), joint neighborhood (all black edges between nodes 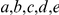), and an exclusive and a common neighbor (black, dotted edges). For each of them we also added their normalized value. Normalization was done by the number of possible edges between the neighbors they have.

To predict whether two non-members 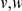 are connected, we compute 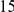 topological graph features of the network around 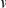 and 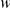 in 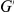 on which the prediction is based. We deduce the features from relational knowledge because the data at hand is anonymized and therefore no node attributes are available. The exact choice of features is rooted in the known structural properties of (online) social networks [30], [31]. The intuition that two people sharing common friends are likely to be friends themselves motivates including a feature that counts the absolute number of common neighbors 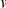 and 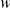 have. However, the absolute number of common neighbors might be misleading if 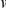 has just a few neighbors, while 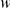 has many. Thus, we add three normalized versions of the number of common neighbors where the normalization is done by the smaller degree, the larger degree and the number of nodes which are neighboring at least one of the two nodes (the so-called *Jaccard coefficient*). The typically high assortativity (measuring the likelihood for nodes to connect to other nodes with similar degrees [32]) and the significant local clustering [33] of nodes in online social networks justifies focusing on the average degree and the clustering coefficient of the common neighbours of 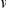 and 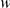. The community structure of social networks [34] leads us to construct several features that reflect the interconnectedness of the member side neighbors of the two nodes as illustrated by Figure 4. Finally, we count the absolute number of distinct paths between 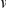 and 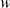 in 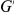 with exactly three edges. For a precise description of the features see Text S2.

For each pair of non-members these scalars are stored in a 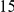 dimensional feature vector. A feature vector relating to two connected non-members is called a positive sample, and one describing unconnected non-members is a negative sample. Based on this vector, supervised machine learning is used to predict which pairs of non-members are connected (acquainted) and which ones are not.

### The Prediction Algorithm

Supervised learning requires a training set on which the classifier’s parameters are adjusted. Its performance is then evaluated on an independent test set. We restrict our predictions to those pairs of non-members with at least one common neighbor among the members. In this respect we follow similar approaches which restricted link predictions to pairs of nodes with a maximum distance of two [9], [12]. Our focus is thus on predicting whether two non-member friends of a member are friends themselves, i.e. whether a pair of non-members is contained as an edge or not. We employ the random forest classifier [35], an ensemble of decision trees that has previously been used for link prediction in dynamic networks [9], [11], [12]. For a more detailed description see Text S3.

Once the random forest has been trained it can be applied to the test set, and edges with a probability higher than some threshold are predicted to exist. This prediction can then be compared to the ground truth.

### Accuracy Measures for Prediction

A good classification result is characterized by a high sensitivity (probability of predicting an edge that truly exists) and high specificity (probability of predicting the absence of an edge that truly doesn’t exist). In the following we use two classic accuracy measures for the link prediction problem, the 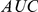 and the 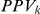 which combine sensitivity and specificity [13], [36]: Varying the threshold allows to trade-off sensitivity vs. specificity. The receiver operating characteristic (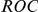 curve) shows the 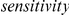 against 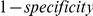 plot. The area under this curve (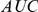) is a scalar performance measure that aggregates the prediction accuracy over all possible settings of this threshold. A perfect predictor achieves an 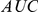 of 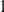 while random guessing in a two-class problem yields a value of 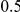.

While the 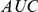 measures the accuracy over the full range of possible thresholds, the 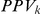 is based on a specific threshold: let 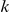 denote the number of positive samples in the test set, i.e., the non-member pairs connected by an edge, and let all samples of the test set, i.e., all non-member pairs having at least one common member friend, be ordered non-increasingly by their prediction value. The 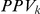 introduced by [11] is defined as the percentage of correctly classified positive samples among the first 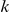 samples in the ranking, and is thus also equal to the sensitivity achieved by predicting these 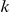 samples to be edges. It can be shown that the specificity is linearly dependent on 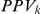 and thus both measures are captured by it. The higher the value the more the number of positive samples is enriched among the 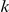 highest-ranked samples. Note that the 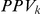 should always be at least as large as the overall fraction of positive edges among all edges. Otherwise, the prediction algorithm performs worse than a naive algorithm in which 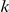 samples are drawn uniformly at random from all samples and predicted to be edges.

**Figure 5:**
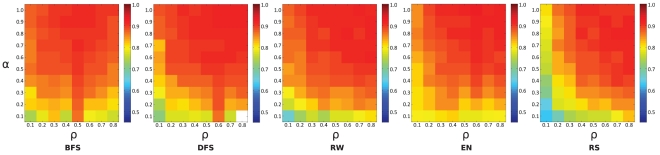
Prediction accuracy (*AUC*) of samples based on all member recruitment models in the cross-validation training scheme applied to UNC data. The white square denotes a data point where there was not enough data to make the prediction.

**Figure 6:**
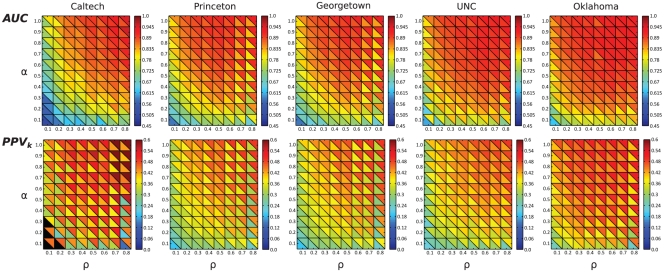
4 → 1 cross-prediction accuracy. Minimal (lower triangle) and maximal (upper triangle) prediction accuracy for all five member recruitment models are shown as a function of platform penetration 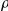 and the disclosure parameter 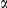. Upper row: 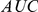; lower row: 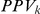; black triangles denote data points where 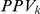 was smaller than the according fraction of positive samples among all samples.

**Figure 7:**
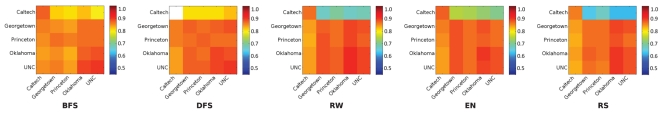
1 → 1 cross-prediction accuracy. 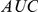
 values for each of the five member recruitment models at 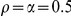. The 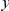 and 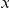-axis show on which network the random forest was trained and tested, respectively. The white field indicates that there were too few edge samples to reasonably train the classifier.

### Training Schemes

All prior work on link prediction with high imbalance between possible and realized future edges that we are aware of uses cross-validation where training and testing are achieved within a single network. In reality, however, one would want an approach that can be trained once and then allows to predict edges in independent networks (cross-prediction). In general, cross-validation within a single network tends to be overly optimistic in its results since training and test samples may be dependent. It is thus more meaningful to test the cross-prediction performance of a prediction algorithm. Accordingly, we have devised the following training scheme (see Text S3):

**1.** **cross-prediction** 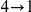 **:** The classifier was trained on subsets of the samples from four of the five data sets and the test set consisted of all samples from the fifth data set. This is an instance of cross-validation across networks, rather than within a network. To stress this difference, we use the term cross-prediction in the following. To be able to compare classification results obtained on different data sets and for varying parameters 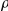 and 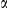, we subsampled the available training data in the following way: First, 1200 positive samples and 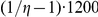 negative samples were selected at random. Here, 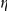 denotes the fraction of positive samples among all samples. To obtain balanced training sets when constructing individual trees of the random forest, we then randomly selected 1200 samples from both classes from the reduced set. We applied this procedure to each of the four training data sets to obtain a total of 4800 positive samples and the corresponding number of negative samples. Since especially the Caltech data set did not always provide enough samples, we oversampled the available data whenever necessary (that is by sampling with replacement). Each tree was trained with a balanced data set of 4800 samples from both classes.

**2.** **cross-prediction** 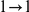 **:** For each pair of data sets, the classifier was trained on one and evaluated on the other. The same sampling scheme was used as above and the trees were trained with 1200 samples per class.

The 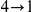 training scheme is less prone to overfitting by training on four different networks, while the 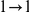 scheme was used to find out whether a single known network contains enough information to obtain high-quality predictions.

## Results

For all member recruitment models, the ratio between the number of positive and negative samples is very small and lies between 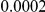 and 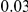 for four out of five of the university networks. This imbalance of the two classes is in the typical range of imbalances for various link prediction problems [9], [11], [36], [37] and seems to determine the hardness of the problem.

As argued above, the prediction accuracy of an intra-network cross-validation should upper-bound the prediction accuracy of an inter-network cross-prediction approach. Thus, as a base line, Figure 5 visualizes the prediction accuracy as measured by the 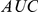, using different member recruitment models in conjunction with cross-validation on the UNC data set. The general pattern is that the prediction accuracy increases with 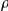 and 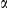. In other words, the greater the percentage of members and the higher their propensity to share their email contacts, the easier it is to predict the network between non-members. One exception to this pattern is the BFS model whose prediction accuracy shows a maximum for 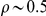. The behavior of the 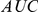 and the 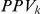 are very consistent over all member recruitment models for all university data sets, implying that the exact model of the member recruitment model is not crucial (see Figures S4 and S5).

The 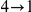 cross-prediction training scheme results in 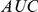 values of at least 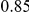 for all combinations with 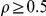 and 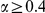 in the case of UNC, Princeton, Georgetown and Oklahoma, for all but the BFS member recruitment model. Similarly, the 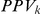 is at least 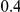 for the same range of 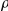 and 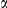, on UNC, Georgetown and Oklahoma, and for all but the BFS and the DFS member recruitment models. That means, if for each data point we select the 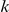 samples with the highest prediction values, at least 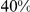 of them indeed represent two non-members that know each other. Figure 6 shows for each combination of 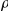 and 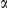 and all five data sets the minimal (lower triangle) and maximal (upper triangle) 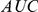 and 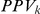 value over all member recruitment models. For the complete results refer to Figures S6 and S7. It can clearly be seen again that the differences depending on the member recruitment model are small in most cases. The one part where the difference is most pronounced is for 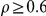 for Georgetown and Princeton, where the BFS member recruitment model results in notably worse predictions than the other member recruitment models.

The 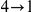 setting is more robust against overfitting since it learns from four independent networks. To see if one can reliably predict with even fewer training data, we also evaluated in the 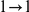 setting how good the predictions remain if the random forest is trained on only one network at 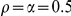. For the case of Facebook in particular, the estimates of 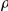 and 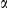 appear conservative, because of the pervasiveness that this platform has achieved, and the simplicity with which members can upload their full email address book. At the time of writing, Facebook still asks novice members, upon the creation of their account, to supply the password of their email provider to parse all their email contacts. The choice of 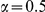 is supported by an informal survey among 100 members of Facebook. Additionally, for the particular case of Facebook, the number of members whose email contacts are known can be expected to further grow with the integrated email service that has recently been introduced. Figure 7 shows the prediction accuracy in the 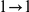 training scheme. On the diagonal, the cross-validation value is plotted as a reference and it can be seen that the 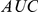 values in this cross-prediction training scheme are around the same value as those of the cross-validation within the same network. It can also be seen that some data sets are easy to predict, namely Oklahoma and UNC, while Caltech is hard to predict based on any of the four other data sets. Furthermore, if the classifier is trained on Caltech data, the predictions are consistently the worst among all cross-predictions. Based on the network statistics shown in Figure 2, it can be seen that UNC and Oklahoma are the two largest networks while Caltech is the smallest, with around half the average degree of the other four networks. At the same time, its clustering coefficient is almost twice as large as that of the others. Thus, the prediction accuracy as measured by 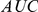 is almost as good in 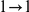-cross prediction as in within-network cross-validation, provided the network used for training is structurally not too different from the one to be predicted.

In summary, we achieve an 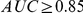 and a 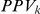 of more than 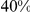 in a cross-prediction training scheme for realistic parameter values of 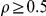 and 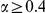. The high 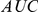 value implies that the prediction is considerably better than random guessing. The 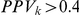 means that, if there is an estimate on the number 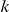 of edges to expect between all pairs of non-members that share a member friend, then 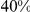 of the predicted edges are indeed edges and of these edges between non-members 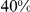 are predicted. Note that the restriction to predicting only connections between non-members sharing a member friend leaves us with at least 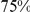 of all befriended non-members (in almost all cases this percentage is above 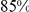, Table S1).

One final remark concerns the variability of prediction accuracy among the member recruitment models. We found that the prediction accuracy is rather independent of how individuals decide to become a member of an online social network platform, but that some percentage of independent decisions like in the 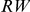, 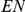, and 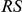 model helps the platform to explore the latent social network more efficiently.

## Discussion

The above results give us a good indication of how accurately one can predict links between non-members of a social network platform, based only on information extracted from the friendship and email contact information of their members. Additionally, the quite high prediction accuracy achieved in inter-network cross-prediction as compared with intra-network cross-validation is astonishing. As always, machine learning can only work if the training data is representative of the observations for which predictions are required in the future. This expectation is borne out by our experiments which show that cross-prediction works best if training and test set are similar in terms of basic network statistics.

Note that we used purely topological or structural indicators, i.e., we only exploited the presence or absence of edges in the computation of features for prediction. Social network platform operators, however, typically have access to much more detailed information on nodes such as the age, sex and (approximate) location of their members; and if they provide messaging services they can infer the quality of an acquaintance from its communication pattern. Including such information into the features will likely improve prediction accuracy.

The ground truth imputation on which the results are based relies on three important modeling decisions which need to be discussed. First, we imply that non-members are similar to members in terms of the revealed network characteristics. Two studies from 2006 and 2009 indicate that there are indeed statistically significant differences between members and non-members among university students: these differences concern age, ethnicity, and gender, but not important social factors such as life satisfaction, social trust, or privacy concerns [38], [39]. Although the sociability of members and non-members was not directly assessed, these studies give no indication that members and non-member differ significantly in the structure of their contact networks. Second, since the contact network between members and non-members of no social network platform is available, we take the known Facebook friendship network as a proxy for the structure of the email contact network between members and non-members. This is justified by the fact that both belong to the large set of social networks with scale-free degree distribution, high clustering coefficient, small-world behavior, and a positive assortativity [31], [40], [41]. Third, we only take into account pure member recruitment models which might not be realistic apiece. The surprising result that the choice of member recruitment models does not alter the main conclusions shows that the analysis of the pure models does not constrain the approach. Even for BFS, which makes good predictions hardest, good results are obtained. This implies that however individuals decide to join an online social network, the unilateral declaration by members of their contacts with non-members allows social network platforms to gain substantial insight into the relationships of non-members. This increase in coverage thanks to link prediction will be most successful if the individual members’ decisions to join the network exhibit some independence - a knowledge that could be exploited by the platform when elaborating new recruitment strategies.

Altogether, our results indicate that knowledge of the social network between members of a platform along with part of the contacts to non-members is sufficient to infer a substantial part of the network between non-members. This perhaps surprising finding has been afforded by leveraging relational information provided by members in a unilateral, non-consensual manner. Ultimately, it evokes the question of the ownership and exploitation of relational data in the information age.

## Supporting Information

Figure S1 **Some structural properties of the networks resulting from applying different member recruitment models on the** Oklahoma **data set in dependence of** ***ρ*** **for** ***α  =*** **1.**
(EPS)

Figure S2
**The coverage of all member recruitment models in dependence of** ***ρ*** **for the networks of the five universities.** For all member recruitment models and all data, 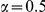, i.e., half of the members have shared their email contacts.(EPS)

Figure S3
**The coverage of all member recruitment models in dependence of** ***α*** **for the networks of the five universities.** For all member recruitment models and all data, 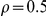, i.e., half of the nodes are selected members.(EPS)

Figure S4
**Shown are the** ***AUC*** **values for all** ***ρ*** **−** ***α*** **combinations in all five data sets.** The random forest was trained on 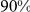 of the samples within the same data set and tested on the remaining 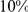 (9/10-cross validation). The procedure was repeated 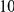 times with randomly chosen training sets, and the values of all runs were averaged. White squares denote data points where there were not enough edge samples to do the learning.(EPS)

Figure S5
**Shown are the** ***PPV_k_*** **values for all** ***ρ*** **−** ***α*** **combinations in all five data sets.** The random forest was trained on 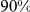 of the samples within the same data set and tested on the remaining 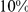 (9/10-cross validation). The procedure was repeated 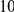 times with randomly chosen training sets, and the values of all runs were averaged. White squares denote data points where there were not enough edge samples to do the learning; black squares denote data points where 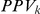 was smaller than the according fraction of positive samples among all samples.(EPS)

Figure S6
**Shown are the** ***AUC*** **values for all** ***ρ*** **−** ***α*** **combinations in all five data sets.** To predict samples from any of the data sets, the random forest was trained on 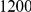 samples each of the other four data sets (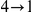 prediction). White squares denote data points where there were not enough edge samples to do the learning.(EPS)

Figure S7
**Shown are the** ***PPV_k_*** **values for all** ***ρ*** **−** ***α*** **combinations in all five data sets.** To predict samples from any of the data sets, the random forest was trained on 1200 samples each of the other four data sets (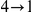 prediction). White squares denote data points where there were not enough edge samples to do the learning; black squares denote data points where 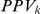 was smaller than the according fraction of positive samples among all samples.(EPS)

Table S1
**Percentage of edges between two non-members that commonly know at least one member of all edges between two non-members.** Shown is the average of ten runs for 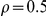 and 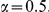

(PDF)

Text S1 **Details about the experimental setting.**
(PDF)

Text S2 **Details regarding feature extraction.**
(PDF)

Text S3 **More information on the learning procedure with random forests.**
(PDF)
