# Correction: One Plus One Makes Three (for Social Networks)

**DOI:** 10.1371/annotation/c2a07195-0843-4d98-a220-b1c5b77a7e1a

**Published:** 2012-04-26

**Authors:** Emőke-Ágnes Horvát, Michael Hanselmann, Fred A. Hamprecht, Katharina A. Zweig

There was an error in the first author's name. The correct spelling is Emőke-Ágnes Horvát. 

