# Supplementary material for: One Plus One Makes Three (for Social Networks)
Source: PLoS One. 2012 Apr 6;7(4):e34740. doi: 10.1371/journal.pone.0034740 (PMC3321038; doi:10.1371/journal.pone.0034740)
Supplement: Table S1 — Percentage of edges between two non-members that commonly know at least one member of all edges between two non-members. Shown is the average of ten runs for and (PDF) [file pone.0034740.s008.pdf]

Table S1

	Caltech	Princeton	Georgetown	UNC	Oklahoma
BFS	92.65	80.12	76.18	75.93	83.63
DFS	93.45	89.06	88.76	80.87	83.82
RW	96.01	93.90	92.12	86.34	90.86
EN	93.10	89.11	88.22	80.67	83.42
RS	95.44	93.66	92.26	86.81	90.94
