# Supplementary material for: One Plus One Makes Three (for Social Networks)
Source: PLoS One. 2012 Apr 6;7(4):e34740. doi: 10.1371/journal.pone.0034740 (PMC3321038; doi:10.1371/journal.pone.0034740)
Supplement: Text S1 — Details about the experimental setting. (PDF) [file pone.0034740.s009.pdf]

Text S1

Modeling the acquisition of new members of social network platforms

To our knowledge there is no exhaustive research on how people decide to become a member of a given social network platform. It is likely to be a mix of independent decisions, especially in the early phase of a new social network platform, and of decisions that depend on the decisions of friends who are already members of the same social network platform. Since there is no model for how people decide on their membership we have implemented five possible scenarios that range from the pure independence model to models with various degrees of independent and dependent decisions, called *member recruitment models*:

1. **Breadth First Search (BFS):** The first person to join the network is chosen uniformly at random. Then all her friends join, after that all their friends and so on. This model is computed by a typical graph search procedure called *breadth first search* [1]. If there is no further friend to join the network, the procedure starts all over with a new person, chosen uniformly at random from all persons that are not yet assigned as members.

2. **Depth First Search (DFS):** The first person to join the network is chosen uniformly at random. Whenever someone has joined, one of her friends will join next. If there is no more friend of the last person to join the network, the search tracks back to the next-to-last person who joined the network and who still has another friend to join it. This model is computed by a typical graph search procedure called *depth first search* [1]. If there is no further friend to join the network, the procedure starts all over with a new person, chosen uniformly at random from all persons that are not yet assigned as members.

3. **Random Walk (RW):** The first person to join the network is chosen uniformly at random. Whenever someone has joined, one randomly chosen friend of her will join next, just as in the DFS. If, however, a person is chosen who already is a member the search stops and the process starts again with a new person, chosen uniformly at random from all persons that are not yet assigned as members. Thus, in general RW produces smaller connected subnetworks of people to join the network.

4. **Ego networks (EN):** A person is chosen uniformly at random and she and all of her friends join the network. The procedure is repeated by choosing a person uniformly at random from all persons that are not yet assigned as members.

5. **Random selection (RS):** Every person joins the network with the same probability.

Next, we introduce the parameter ρ that describes the percentage of members of a real social network that are also members of an online social network. This parameter is modeled in 8 discrete steps from $\{0.1, 0.2, 0.3, 0.4, 0.5, 0.6, 0.7, 0.8\}$. Each of the procedures is then applied until the desired number of people $\rho|V|$ is assigned as members.

Modeling the contact sharing behavior of members

Our final parameter α is related to the number of people sharing their address books with the social network platform. An α value of 1 means that each member (100%) has shared all her email contacts, a value of 0.9 means that 90% of the members have shared all of their contacts, etc. We show results for $\alpha \in \{0.1, 0.2, 0.3, 0.4, 0.5, 0.6, 0.7, 0.8, 0.9, 1.0\}$.

Structural properties of partitions from different member recruitment models

Not surprisingly, the member recruitment models produce different partitions of the same underlying graph, with different structural properties. Figure S1 shows the dependence of the following six structural properties on ρ and the member recruitment model applied to the `Oklahoma` data set:

1. The number of edges within the member set.
2. The average degree of members in the within-platform network, i.e., the average number of edges to other members.
3. The number of edges between members and non-members. In general it depends on α which was set to 1.0 in this case.
4. Average degree of the members, average number of edges to members and non-members.
5. The average number of edges of a non-member to members.
6. The average clustering coefficient within the member-to-member network. The *clustering coefficient* of a node is defined as the probability that any two of its neighbors are connected by an edge. For all member nodes their clustering coefficient in the member-to-member network is computed and averaged.

The number of edges within the member set increases monotonically with ρ as to be expected, but the average degree of the members peaks at $\rho = 0.5$ for BFS, DFS, and EN, finally converging to the average degree in the network of 102. The number of edges between members and non-members peaks for all five member recruitment models, but at different ρ-values. The reason for this is that for all methods but RS those nodes with a higher degree have a higher chance to get added to the member set than those with a small degree. This can also be seen from the fact that the remaining nodes are more likely to have small degrees and thus a smaller number of edges to the members as well. Moreover, this manifests in the average total degree of the members which is quite consistently 100 in the case of RS and but is much higher for lower ρ for all other methods. For the same reason, the average degree of non-members rises linearly with ρ in the RS model since there is a linear increase in members. For all other models, low-degree nodes have a higher probability to be non-members. Consequently, for increasing number of members and ever smaller degree of non-members, the average degree of the non-members peaks at some ρ-value. As to be expected, when sampling from a social network with a high clustering coefficient, BFS and EN results in member-to-member networks with a high average clustering coefficient, while random sampling consistently results in the lowest average clustering coefficients for $\rho < 0.4$. For $\rho \geq 0.4$ all clustering coefficients fluctuate around the average clustering coefficient of the full data set, which is 0.23.

Another important structural measure is the fraction of *relevant* non-members among all members, i.e. those which are connected to at least one of the members. This property depends on the member recruitment model X, the platform penetration value ρ, and the disclosure parameter α, and is defined as:

$$coverage(X, \rho, \alpha) = \frac{|\overline{M}_R|}{|\overline{M}|}, \tag{1}$$

In Figure S2 and Figure S3 we show the coverage for all member recruitment models and all five university data sets:

1. in dependence of ρ with $\alpha = 0.5$ (Figure S2), and
2. in dependence of α with $\rho = 0.5$ (Figure S3).

The choice of $\alpha = 0.5$ respectively $\rho = 0.5$ is reasonable (see the **Results** section of the main article). Figure S2 shows that for the selected interval of $\rho \in [0.1, 0.8]$ and $\alpha = 0.5$ the coverage is larger than 0.7 in all cases and larger than 0.8 in the majority of member recruitment, ρ-value and data set combinations. Note that the coverage drops for BFS, DFS, and EN for $\rho = 0.8$. The same three recruitment models yield smaller coverages when fixing $\rho = 0.5$ and exploring the entire range of α values (Figure S3). In comparison, the coverage is more sensitive to the disclosure parameter α than to the platform penetration value ρ.

References

1. Cormen TH, Leiserson CE, Rivest RL, Stein C (2003) Introduction to Algorithms. MIT Press.
