# Supplementary material for: One Plus One Makes Three (for Social Networks)
Source: PLoS One. 2012 Apr 6;7(4):e34740. doi: 10.1371/journal.pone.0034740 (PMC3321038; doi:10.1371/journal.pone.0034740)
Supplement: Text S2 — Details regarding feature extraction. (PDF) [file pone.0034740.s010.pdf]

Text S2

Ground truth

To test a *prediction* result obtained from any kind of machine learning approach, ground truth data is required, i.e. data for which the true outcome is known. To avoid bias, this data should be independent from the training data. In our setting, we know the "real" social network as defined by the underlying graph G. We then split the node set into two sets of interest, namely M and $\overline{M}_R$. These two sets induce three different kinds of edge sets, which are all subsets of E: E_I, the connections between members, E_B, the edges between exactly one member and one relevant non-member, and the network between non-members.

Only the first and the second network are used for calculating the features, and based on their structure, we predict whether two given relevant non-members v and w have an edge. Since we know the third network, the prediction can be labeled as *true positive*, if the edge is predicted and it exists in the third network, as *true negative* if it is predicted not to exist and it does not exist in the third network, as *false positive* if predicted but non-existent, and finally as *false negative* if not predicted but existent. As it is typical for social networks, the university networks that we have worked with are quite sparse. Thus, most of the edges between non-members do not exist and it would be by far the most efficient way to just predict 'no' for each possible edge since this would result in correct predictions for almost all cases. We are, however, much more interested in finding out who is connected than in who is not, and would rather have some false positives than no predictions at all on the class of interest. This is a well-known trade-off between the *specificity* [fraction of correctly identified negatives] and *sensitivity* [true positives which are correctly identified as such] (see Supporting Information S3).

To predict whether two non-members v and w are connected, we compute certain topological properties based on their connections to members and on the connections between the members they know. These so-called *features* are described in the following.

Features

We have used 15 different features to describe the local network environment between two non-member nodes v, w. Recall that $n(v)$ denotes the number of neighbors of node v.

1. **Absolute number of common neighbors**. Note that these are members by definition:

$$|n(v) \cap n(w)| \tag{1}$$

2. **Absolute number of common neighbors, normalized by the minimum of the two degrees**:

$$\frac{|n(v) \cap n(w)|}{\min\{deg(v), deg(w)\}} \tag{2}$$

3. **Absolute number of common neighbors, normalized by the maximum of the two degrees**:

$$\frac{|n(v) \cap n(w)|}{\max\{deg(v), deg(w)\}} \tag{3}$$

4. **Jaccard coefficient:** Absolute number of common neighbors, normalized by the number of nodes which are neighbor of at least one of the two nodes:

$$\frac{|n(v) \cap n(w)|}{|n(v) \cup n(w)|} \tag{4}$$

5. **Average degree of common neighbors** where the degree $deg_I(u)$ of neighbor u is given by its number of edges to other members:
$$\frac{\sum_{u\in n(v)\cap n(w)} deg_I(u)}{|n(v)\cap n(w)|} \tag{5}$$

6. **Average clustering coefficient of the common neighbors**. The *clustering coefficient* $cc(u)$ of a member u is defined as the number of edges $e_I(u)$ between other *members* it is connected to, divided by the possible number of these edges:
$$cc(u) = \frac{e_I(u)}{deg_I(u)(deg_I(u)-1)/2}. \tag{6}$$
The average clustering coefficient of common neighbors is defined as the arithmetic mean of all common neighbors of v and w:
$$\frac{\sum_{u\in n(v)\cap n(w)} cc(u)}{|n(v)\cap n(w)|}. \tag{7}$$

7. **Absolute number of edges between common neighbors**: see black, dashed edges in **Figure 4** (main text).

8. **The former, normalized by the possible edges between common neighbors**.

9. **Absolute number of edges between the exclusive neighbors of x and y**: see black, straight edge in **Figure 4** (main text).

10. **The former, normalized by the possible edges between exclusive neighbors**.

11. **Absolute number of edges in the joint neighborhood of v and w**: see all black edges between nodes a, b, c, d, e in Figure 4 (main text).

12. **The former, normalized by the possible edges in the joint neighborhood of v and w**.

13. **Absolute number of edges between an exclusive and a common neighbor**: see black, dotted edges in **Figure 4** (main text).

14. **The former, normalized by the possible edges between exclusive and common neighbors**.

15. **Absolute number of paths of length 3 between v and w**.

The two nodes v, w in Figure 4 from the main text have the following feature vector:
$[3, 3/4, 3/5, 3/6, 8/3, (2/3 + 2/3 + 1)/3 = 7/9, 3, 3/3, 1, 1/3, 5, 5/15, 2, 2/9, 6]$.

For each pair of non-members, these features can be computed (although they might be zero for many of them) and stored in a vector with 15 dimensions. The combination of such a vector and a *label* that indicates whether the two non-members are indeed connected (1) or not (0) is called a *sample*. It turned out that those pairs of non-members that were indeed connected but did not share at least one friend in M [first feature] often had 0-values in all other dimensions as well. For 0-vectors it is not possible to learn any distinguishing features and thus we restricted our predictions to those samples with a non-zero entry in the first dimension. Note that the rest of the dimensions might still be 0. A similar aspect has been reported in [1], where predictions on the existence of an edge are differentiated by the distance the nodes have disregarding this edge. In our case, we restrict ourselves to those nodes with distance 2. Table S1 shows for $\rho = \alpha = 0.5$ the percentage of those edges between non-members that we regard among all edges between non-members.

The Algorithm

The full algorithm to create the samples for prediction can thus be sketched as follows:

```
1 For each of the five data sets
2   For $\rho$ = 0.1 to 0.9 do
3      For $\alpha$ = 0.1 to 1.0 do
4         For each member recruitment model $P$ do
5            compute $M$ and $\overline{M_R}$
6            for each node $m \in M$
7             for all pairs of neighbors $(v, w)$ of $m$
8               compute the feature vector of $v, w$
9               add information about whether $v, w$ are connected (label)
10               write out IDs of v and w, the vector, and the label
```

Note that lines $6, 7$ guarantee that only samples with a non-zero entry in the first dimension of the feature vector are generated by the algorithm. For all of the $5 \cdot 9 \cdot 10 \cdot 5$ possible combinations of the five data sets, 9 ρ-values, 10-α-values, and the member recruitment process we have written out the feature vectors for all possible samples with a non-zero first entry and used this entire set to subsample in the following machine learning approach.

References

1. Lichtenwalter RN, Lussier JT, Chawla NV (2010) New perspectives and methods in link prediction. In: Proceedings of the 16th ACM SIGKDD International Conference on Knowledge Discovery and Data Mining (KDD '10). pp. 243–252.
