# Supplementary material for: One Plus One Makes Three (for Social Networks)
Source: PLoS One. 2012 Apr 6;7(4):e34740. doi: 10.1371/journal.pone.0034740 (PMC3321038; doi:10.1371/journal.pone.0034740)
Supplement: Text S3 — More information on the learning procedure with random forests. (PDF) [file pone.0034740.s011.pdf]

Text S3

Supervised learning requires a training set on which the classifier's parameters are learnt. An independent test set is used to evaluate its performance. For each test sample the classifier returns a vector that estimates the probability that the sample belongs to any of the classes under consideration, in our case edges and non-edges. We employ the *random forest* classifier [1], a state-of-the-art supervised learning approach which was recently successfully used for link prediction [2]. A random forest consists of a set of decision trees. Each node of a decision tree contains a simple test of the form *Is the value of the ith dimension d_i lower than threshold t_i?*, and all leave nodes contain a deterministic class label (in our case: edge, non-edge). Given a test sample, all trees of the forest are traversed from their roots to one of their leaves. Finally, the sample arrives in one of the leaves that, in our case, classifies this sample as an edge or a non-edge sample. The number of trees voting for a certain class are counted and the percentage of trees classifying the test sample as an edge defines its prediction value. The random forest is built (learnt) on a given set of training samples. For each tree, the following procedure is repeated: We start with the root node that contains all samples. At each node, the feature that best splits the samples in the node is selected among a randomly chosen subset of size $mtry$ of the P available features and an adequate threshold is found. Subsequently, the node is split and the process is recursively repeated for each of the resulting subsets until a pure subset with samples from only one class is produced. Here we build $ntree = 500$ decision trees. We learn based on $P = 15$ features and at each node of the decision trees, the number of features sampled for splitting is $mtry = \sqrt{P}$.

Learning schemes

To the best of our knowledge, so far authors have only considered cross-validation within single networks but not cross-prediction between networks in the classic link-prediction problem. Cross-validation which randomly partitions the available non-member pairs into training and test samples, can certainly be used to obtain performance estimates. However, the independence of these sets is only guaranteed if training and test samples stem from *different* connected components. Typically, this is not the case such that cross validation can lead to overly optimistic performance estimates. It is thus more informative to train and test the classifier on truly independent sets[1].

To understand the dependence of the prediction accuracy on different learning schemes, we have devised three different training and test set scenarios:

1. intra-network cross-validation: Training and testing within the same network based on *10 fold cross-validation* where 90% of the samples are drawn uniformly at random to construct the training set, and the remaining 10% define the test set; each of the 10 folds was used as the test set once and the results were averaged.

2. inter-network 4 → 1**:** In another setting, the random forest was trained on subsets of the available samples from four of the five data sets and the test set consisted of all samples from the fifth data set.

3. inter-network 1 → 1**:** In the last setting, for each pair of data sets, the random forest was trained on the first data set and the second was predicted.

Imbalance of sample classes

A special aspect of the link prediction scenarios is the ratio of the two classes (positive and negative samples) are extremely imbalanced: for most of our settings, the ratio is about 1 : 50. More specifically,

[1]Note that in a recent paper, Leskovec et al. [3] have applied a cross-prediction approach to a different flavor of a link prediction method in which the existence of an edge is known but the weight of it (liking or disliking) has to be predicted. This flavor of a link prediction problem is different, especially since it shows totally different ratios of both classes.

the minimum ratio is 0.0002 for `UNC` with DFS when $\rho = 0.9$ and $\alpha = 1$; the maximum is 0.0293 for `Princeton` with RS when $\rho = 0.1$ and $\alpha = 0.1$. For `Caltech` the values are, however, one order of magnitude higher: the minimum is 0.0019 with DFS when $\rho = 0.9$ and $\alpha = 0.6$; the maximum is 0.03403 with RS when $\rho = 0.1$ and $\alpha = 0.1$. Since we are interested in both positive samples (edges) and negative samples (non-edges), the random forest is trained with a balanced training set. Therefore, we first randomly subsample the available training data to obtain 1200 edge samples. In the case of `Caltech`, we have to oversample the existing samples for most combinations with $\rho > 0.7$. Then, we randomly select as many negative samples as it requires to preserve the ratio of the two classes. Finally, for each tree of the forest we randomly draw 1200 samples from the selected negative samples to obtain a balanced set. Note that using the same number of training samples for all parametrizations allows us to compare the results. Further note that we use the complete, imbalanced data for testing.

Prediction accuracy measures

A good classification result is characterized by a high detection rate of both positive samples (sensitivity) and negative samples (specificity).

Let S be the set of all test samples, and $s \in S$ be an instance of it. Then $pred(s)$ gives the *prediction value* of the *random forest* based on the set of training samples T. For a given threshold τ, let $Pred_\tau$ denote the set of all test samples s with $Pred(s) > \tau$. Let E_S denote the set of positive samples in S, i.e., those samples of which we know that in G there is an edge between the corresponding v and w. Let NE_S denote the set of negative samples in S. Let $\eta(S)$ denote the positive sample density in S, i.e., $\eta(S) := |E_S|/|S|$. Then, the following accuracy measures of predictions are defined as follows:

1. *Sensitivity*: percentage of correctly identified positive samples.
$$\text{sensitivity} = \frac{|E_S \cap Pred_\tau|}{|E_S|}. \tag{1}$$
$TP := E_S \cap Pred_\tau$ is called the set of *true positives*.

2. *Specificity*: percentage of correctly identified negative samples.
$$\text{specificity} = \frac{|NE_S| - |NE_S \cap Pred_\tau|}{|NE_S|}. \tag{2}$$
$TN := NE_S - Pred_\tau$ is called the set of *true negatives*. It is necessary to evaluate both measures (sensitivity and specificity) at the same time because a high sensitivity can be easily achieved by predicting that all samples are edges, and conversely a high specificity can be achieved by always predicting non-edges.

3. *Receiver Operating Characteristic (ROC curve)*: The ROC curve is computed for various thresholds and the corresponding pairs of sensitivity and false positive rate (1 - specificity), which are plotted against each other. It thus gives the trade-off between a good sensitivity and a low false positive rate at various thresholds.

4. *Area under curve (AUC)*: The area under the ROC curve (AUC) gives the probability that for any pair of a positive and negative sample, the positive sample receives the higher prediction value than the negative sample [4]. A value of 0.5 denotes that the prediction makes blind guesses, a value of 1.0 indicates a perfect prediction.

5. *Positive predictive value (PPV)*: The PPV measures the percentage of correctly classified edges within the set of predicted edges. It is dependent on the positive sample density in the test set, since a high number of negative samples may lead to a large absolute number of false positive detections, decreasing the PPV.

6. PPV_k: The PPV_k is a special case of the PPV that allows to compare the accuracy of a prediction method independently of the edge sample density in the test set. Let E_S denote all samples where the two non-members v and w are connected, i.e., $(v, w) \in E_U$, and $NE_S = S - E_S$. We rank all predictions by their prediction value and accept the first $|E_S| = |E_U|$ ranks as edges. It is clear that in general $|E_S|$ would not be known, but as a accuracy measure it has the advantage that in this special case, the PPV and the sensitivity are identical. Thus, a higher PPV_k is always beneficial, since in this special case $|NE_S| = TN + FP$ and thus $specificity = 1 - (1 - PPV_k)|E_S|/|NE_S|$. In other words, a higher PPV_k always leads to an increase in both sensitivity and specificity.

References

1. Breiman L (2001) Random forests. Machine Learning 45: 5–32.

2. Lichtenwalter RN, Lussier JT, Chawla NV (2010) New perspectives and methods in link prediction. In: Proceedings of the 16th ACM SIGKDD International Conference on Knowledge Discovery and Data Mining (KDD '10). pp. 243–252.

3. Leskovec J, Huttenlocher D, Kleinberg J (2010) Predicting positive and negative links in online social networks. In: Proceedings of the 19th International Conference on World Wide Web (WWW'10). pp. 641–650.

4. Fawcett T (2006) An introduction to ROC analysis. Pattern Recognition Letters 27: 861–874.
